# Unveiling Energy Dynamics of Battery Electric Vehicle Using High-Resolution Data

**DOI:** 10.1038/s41597-025-06148-5

**Published:** 2025-11-28

**Authors:** Mohamed Yasko, Adamou Moussa Issaka, Fanghao Tian, Hussain Kazmi, Johan Driesen, Wilmar Martinez

**Affiliations:** KU Leuven/EnergyVille, Thor Park 8310, Genk, Belgium

**Keywords:** Energy grids and networks, Electrical and electronic engineering

## Abstract

Battery electric vehicles (BEVs) have increasingly positioned themselves as a critical technology in the power system, impacting the world’s energy consumption. Understanding the BEV energy dynamics can contribute to vehicle, infrastructure, and grid optimization. Currently, BEV manufacturers provide limited access to the vehicle’s high energy consuming components, such as the battery and the charger. Therefore, existing public datasets consist mostly of aggregated data collected from charging points outside the vehicle, resulting in lower data resolution and not capturing the actual energy dynamics. This paper fills this dataset gap by developing a data generation method to collect datasets, including the actual energy values for the charger, the battery, and the auxiliary devices, using measurement with a second resolution. The collected dataset illustrates energy dynamics under different modes (charging, driving, parking) and environmental conditions. This dataset provides detailed technical insights that can be used to optimize smart charging, reduce operational costs, understand usage, improve the operation of high energy consuming components, build AI models, and analyze grid impact.

## Background & Summary

Battery electric vehicles (BEVs) represent one of the most efficient and sustainable solutions for electrifying transportation. According to the International Energy Agency’s Announced Pledges Scenario^1^, the global fleet of electric vehicles is projected to reach 580 million by 2035, with passenger light-duty (PLD) BEVs accounting for approximately 75% of this total. This rapid growth will significantly increase the power and energy demands. As a result, collecting large-scale, real-world datasets is crucial for accurately estimating and forecasting BEV demand profiles.

To effectively assess and quantify the actual energy consumption of PLD-BEVs, it is essential to conduct extensive experimental data collection using real BEV models. Such data can provide deeper insights into infrastructure requirements across various use cases such ashome, workplace, public street, commercial, and highway charging scenarios. Moreover, this data is valuable for optimizing charging schedules and reducing energy costs for BEV users and charging station operators.

As indicated in Table 1, existing public datasets^2–8^ on BEV energy use primarily focus on aggregated input AC energy per charging session or station. While such data is useful for estimating total energy consumption per vehicle or charging station using many sessions over long periods, it does not accurately reflect the actual energy delivered to the battery due to conversion losses in chargers and variability among BEV models. These model-specific losses are typically not accounted for in previous datasets.

**Table 1:** Overview of relevant public datasets.

Dataset	Description	Location	Date
High-resolution EV charging data from a workplace setting^2^	Slow charging sessions with total energy per session for various vehicles. Data resolution is unknown.	several sites in the USA	2014–2015
ACN-data^3^	Slow charging sessions with total delivered energy per session across three workplace sites. Data resolution is unknown.	California/USA	2019
One Year Recordings of EV Charging Fleet^4^	Charging data for power quality measurements for energy and harmonics estimation using 10 minutes resolution.	Switzerland	2018–2022
ZTBus dataset^5^	Driving data from electric city bus driving missions including power and energy values at 1 second resolution.	Switzerland	2019–2022
A dataset for multi-faceted analysis of EV charging transactions^6^	Slow charging sessions data from various public places (hotel, restaurant, etc.). Data resolution is unknown.	South Korea	2021–2022
Real-world EV data^7^	Charging data for battery pack including temperature, energy, etc., for a single vehicle. Data resolution is unknown.	California/USA	2022
EV Charging Dataset^8^	Slow charging sessions including total energy per session collected at a parking lot. Data resolution is unknown.	Canada	2024
This dataset^9^	Slow charging, fast charging, driving, and parking sessions using 1-second resolution, including variables such as speed, distance, voltage, current, power, energy, temperature, etc, for charger and battery systems.	Belgium	2024–2025

This paper addresses these limitations by introducing a comprehensive dataset that captures energy consumption at the charger and battery levels for PLD-BEVs during charging (AC slow and DC fast), driving, and parking. The dataset offers high-resolution (1second) time series measurements of current, voltage, power, temperature, state of the charge (SoC), speed, distance, etc. These variables are provided for the charger, battery, and auxiliary devices. The full dataset^9^, collected in Belgium under LV grid and weather conditions, includes data from two BEV users operating two different vehicle models. A subset of this dataset was previously used in research works^10,11^ to evaluate charging efficiency across different BEV models and operating conditions, revealing that low charging power levels can significantly reduce overall energy efficiency.

The main contributions of the present research are as follows:

1. **High-resolution timeseries data**: The dataset provides time-series data at one-second resolution, in contrast to typical session-level datasets that report only aggregated energy values without detailing the temporal distribution of power, SoC, and temperature throughout the session.
2. **Comprehensive data coverage**: Unlike most of the existing public datasets shown in Table 1 that focus only on charging point measurements, this dataset includes detailed parameters from the battery, charger, and auxiliary devices, offering a holistic view of the BEV’s energy dynamics.
3. **Multiple operational modes** : The dataset covers all BEV operating modes, including slow charging, fast charging, driving, and parking, making it suitable for various analysis scenarios.
4. **Reproducible data acquisition**: The data were collected using a CAN bus-based method which is capable of interfacing with multiple BEV models. Python scripts are provided to convert raw MF4 files into CSV format, enabling reproducibility and extension by other research works.

The collected dataset has broad applications in charging profiling, optimal charging system design, energy modeling, and infrastructure planning. As summarized in Table 2, it can benefit a wide range of stakeholders–including charger and battery system designers, charging station operators, BEV manufacturers, smart charging developers, and academic researchers–by offering detailed insights into real-world BEV energy dynamics across relevant sessions such as charging, driving, and parking.

**Table 2:** Potential users of the collected datasets.

Potential Users	Possible Benefits	Session
Charger designers	The battery voltage, current and temperature profiles can provide realistic load profile for optimizing control and design of AC and DC chargers	Charging
Battery systems designers	All the collected battery and chargers values can provide insights into optimal module/cell balancing and battery management systems	All the sessions
Charging system operators	The actual energy consumption can provide insights into predicting actual energy and optimize the charging operation for different BEV models and the charging station	Charging and parking
EV models manufacturers	The actual energy consumption during the charging, driving and parking under different operating conditions (e.g., temperature) can provide insights into further optimization of battery, charger, auxiliaries, etc.	All the sessions
Smart charging algorithms developers	The actual energy consumption results can be used to develop better smart charging strategies taking into account the auxiliaries consumption while parking for long time under certain weather temperature	Charging and parking
Academic researchers	The dataset can be used for energy modeling, AI model building, grid impact analysis, etc.	All the sessions

## Methods

This section presents the developed data generation method, including both data collection and processing. The data collection covers the BEV model specifications, measurement devices, and data logging device. The data processing workflow is then detailed, including preprocessing, classification, cleaning, and storage in the research data repository.

### Data Collection

**Fig. 1:**
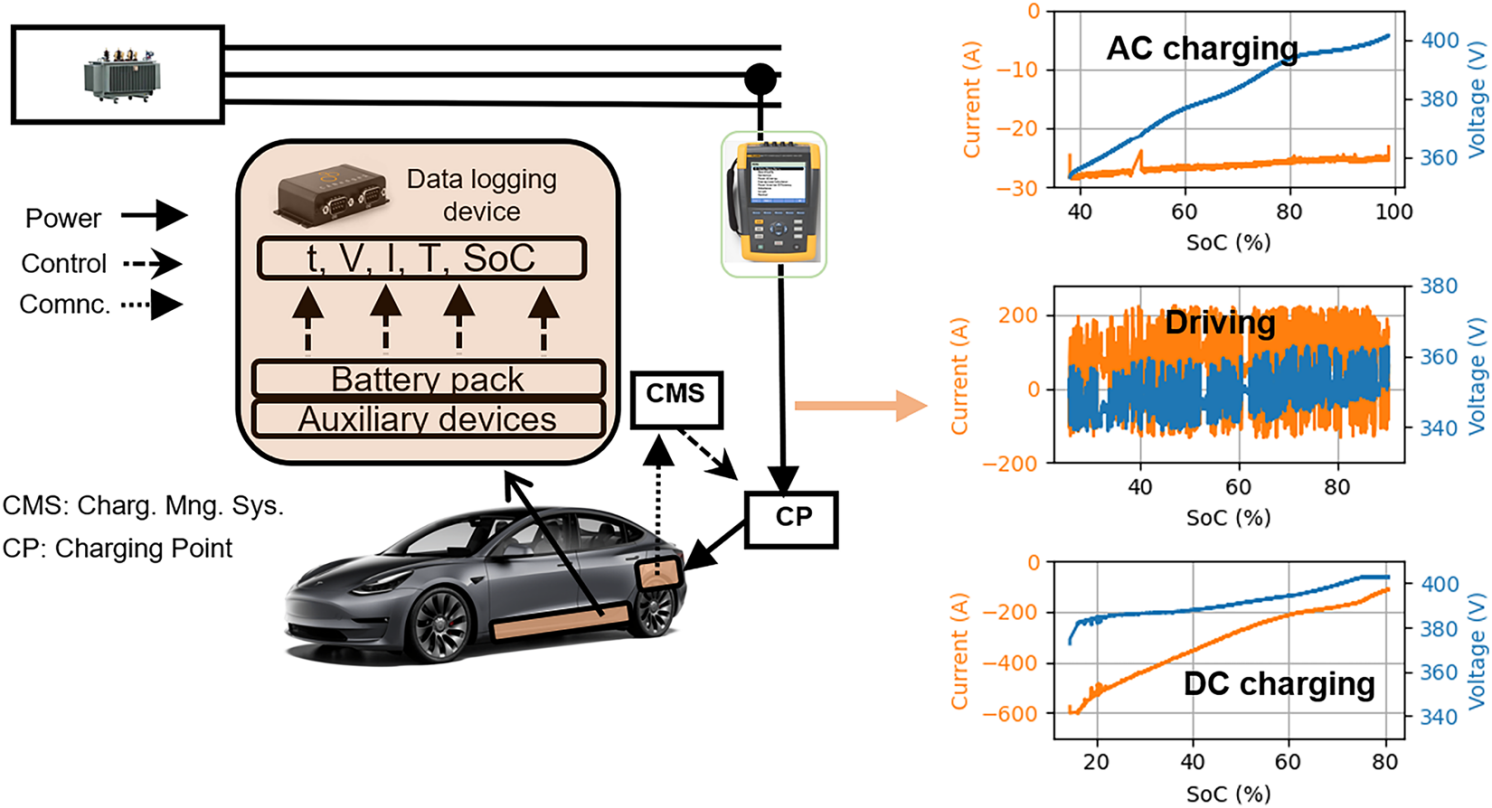
Data collection setup and example of battery profiles.

In this research work, we developed a comprehensive measurement, communication, and control system using out-of-the-shelf data logging^12^, control and measurement device^13^ as illustrated in Fig. 1. This data logger is a plug and play device that records timestamped CAN data to an extractable SD card in MF4 format. This data generation method can be used to collect high-resolution datasets from most of the BEV models as it is based on reading data from the automotive standard CAN-bus systems. Examples of typical battery voltage and current profiles are illustrated for slow AC charging, driving, and fast DC charging. During slow AC charging (11 kW), the current remains relatively constant at around 30 A, while the voltage gradually increases, reaching approximately 400 V, corresponding to a 400 V battery class. In contrast, fast DC charging (250 kW) begins with a high current–often around 600 A–rapidly decreasing as the voltage rises and transitions to a constant voltage phase. During driving, both voltage and current fluctuate significantly, reflecting dynamic energy demands and regenerative braking events that involve both discharging and recharging phases.

Control signals for different operating conditions are sent from the central management system (CMS) using the open charge point protocol (OCPP), the universal open communication standard between the charging point (CP) and the CMS. All sessions were recorded using the same data logging device^12^ to ensure consistency. Additionally, controlled experimental tests were designed to extend the dataset with measurements across a broad spectrum of power and battery SoC collected over different days and seasons.

This dataset comprises charging sessions conducted at various public locations, including on-street parking, workplaces, and other accessible sites. The driving sessions cover highway and urban environments, capturing diverse usage conditions. Each charging session includes a range of operational characteristics, offering a comprehensive representation of real-world charging behavior.

The collected dataset consists of second-resolution data for BEV models utilizing LFP and NCA battery technologies. The BEV models are Tesla model Y standard range (SR) and model Y long range (LR). This model difference allows comparison and highlights the energy consumption due to driving habits, environmental conditions, and vehicle characteristics. These vehicle models are the most popular models in the European market^14^. Table 3 summarizes the specifications of these models, including their initial battery energy capacities–60.5 kWh for BEV1 and 78.8 kWh for BEV2. The operational periods start from 23-02-2023 for BEV1 and 29-09-2022 for BEV2, respectively. However, the data collection period covers from 23-07-24 to 18-02-25 for BEV1 and 08-05-24 to 30-04-25 for BEV2.

**Table 3:** BEV model specifications.

BEV	Cap. (kWh)	Batt. tech.	Model	Motor type	Motor power (kW)	Start date	Data coll. period
BEV1	60.5	LFP	Tesla Y SR	RWD	220	Feb 2023	23-07-24 to 18-02-25
BEV2	78.80	NCA	Tesla Y LR	RWD	324	Oct 2022	08-05-24 to 30-04-25

### Data Processing

**Fig. 2:**
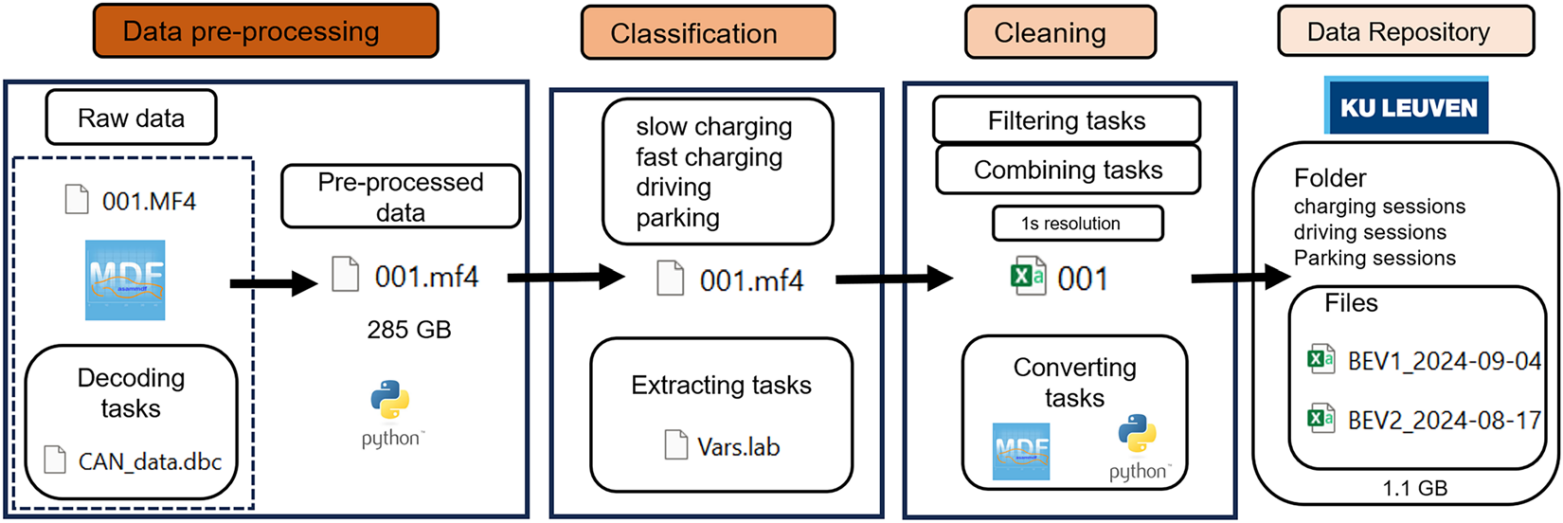
Data processing workflow.

Figure 2 illustrates a structured workflow for processing the collected data from CAN bus logs in MF4 format. The process begins with data pre-processing, where raw data files (001.MF4) are decoded using associated DBC files (CANdata.dbc) to extract interpretable signals, producing a dataset containing pre-processed .mf4 files. This dataset then undergoes classification, identifying relevant operation modes such as slow charging, fast charging, driving, and parking. Classification is supported by extracting relevant variables through task definitions stored in a file like Vars.lab. The third stage, cleaning, focuses on improving data usability: filtering and combining tasks are applied to ensure consistency at a fixed time resolution (1s resolution). During this stage, data is also converted into csv format (001.csv) using Python and MDF tools. Finally, in the data repository stage at KU Leuven research data repository^9^, the cleaned datasets are organized into categorized folders with files named according to specific sessions. The Data Records section of this work further details the data structure and provides file examples as well as Python scripts to reproduce the entire data processing workflow. This systematic approach ensures efficiency, reproducibility, and repeatability for this dataset.

## Energy Consumption Analysis

This section presents the energy consumption analysis focusing on quantifying the losses and consumption patterns for the charger, the battery, and auxiliary devices. This analysis is carried out for four different operating conditions including slow charging, fast charging, driving and parking under different environmental conditions.

### Slow Charging Data

**Fig. 3:**
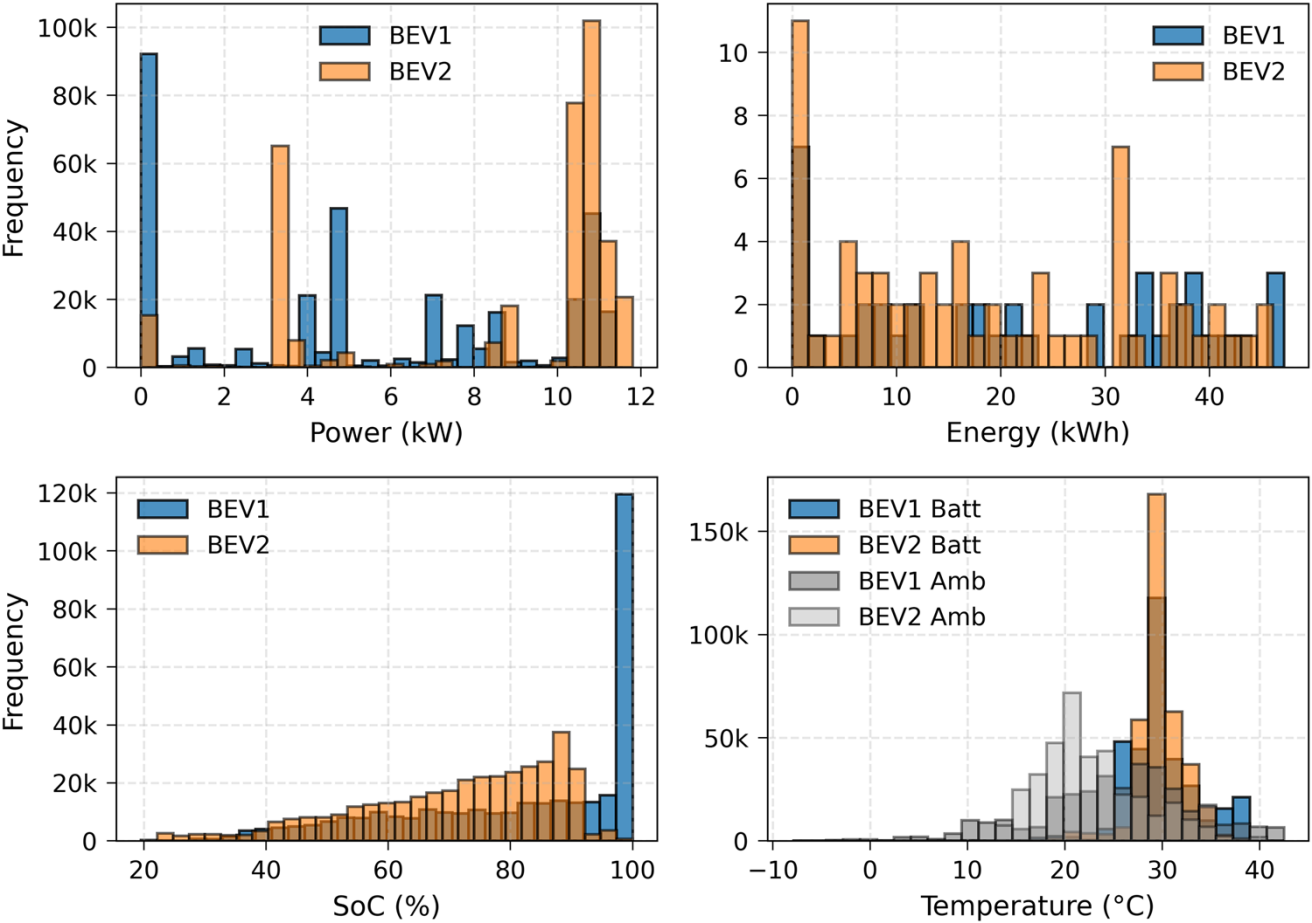
Slow charging dataset distribution.

The dataset collected from the two BEVs for AC or slow charging sessions is illustrated in Fig. 3. This histogram represents the distribution of charging data points regarding charging power, total energy, the SoC, and the battery’s maximum (BEV Batt), and ambient (BEV Amb) temperatures.

The power distribution shows charging sessions collected mostly in the workplace (day charging) and on the street (overnight charging). The workplace charging system uses smart charging algorithms to reduce the power, as can be seen in the main data point frequency for 0 kW (postponing the charging schedule), 4 kW (minimum three-phase power), and 11 kW (maximum power). The other frequent power levels include 1.3 kW (minimum single phase power) and 8 kW (between minimum and maximum possible power levels). The charging power level for street charging is usually around the maximum power level, as smart charging algorithms are not used to control the charging operation.

The energy distribution shows the energy requirement for the two BEVs ranging from 10-40 kWh. This total energy depends on the battery SoC at the beginning of the charging session and the requested final SoC. The SoC distribution is from 40–100% for BEV1 and 40–90% for BEV2 as this user limits the SoC to 90% for battery aging purposes. The battery maximum temperature (BEV batt) distribution shows the temperature profiles during charging sessions which usually ranges from 25 °C-37 °C for BEV1 and 25 °C-35 °C for BEV2. The ambient temperature (BEV Amb) of charging points where the BEVs are parked during charging scenarios is also collected from the vehicle’s temperature sensors. This temperature ranges from 10 °C to 40 °C as most datasets were collected from July to December, and the BEVs were used less during the rest of the months. Additionally, the vehicle entered sleep mode during some sessions, which collected few data points compared to certain sessions with complete resolution data points for the whole duration.

**Fig. 4:**
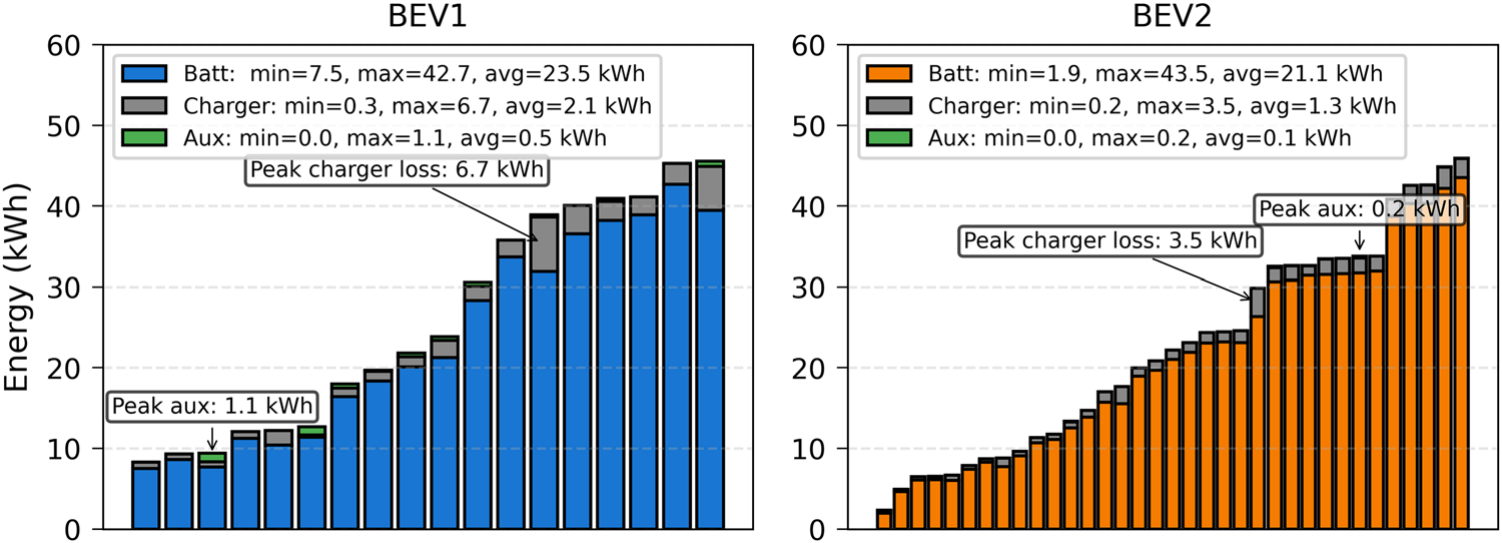
Energy breakdown for selected charging sessions.

The energy breakdown of some selected sessions is illustrated in Fig. 4. The average energy charged by the battery is around 20 kWh, while the maximum values are 40 kWh. During charging, energy losses occur mainly in the charger that converts grid AC power to battery DC power. On average, this charger’s energy loss is around 1-2 kWh, while auxiliary devices consume around 0.1-0.5 kWh. The following energy formulas calculate the losses illustrated in Fig. 4.

1 $${E}_{{\rm{grid}}}=\sum \left({P}_{{\rm{chargeLine}}}\cdot {\rm{\Delta }}t\right)$$

2 $${E}_{{\rm{batt}}}={E}_{{\rm{tot}}\,{\rm{batt}}\,{\rm{ch}}}-{E}_{{\rm{tot}}\,{\rm{batt}}\,{\rm{dch}}}$$

3 $${E}_{{\rm{charger}}}={E}_{{\rm{grid}}}-{E}_{{\rm{batt}}}$$

4 $${E}_{{\rm{aux}}}={E}_{{\rm{tot}}\,{\rm{batt}}\,{\rm{dch}}}$$

where:

- *P*_chargeLine_: AC charging line power [kW, from dataset],
- Δ*t*: charging time step [second, from dataset],
- *E*_grid_: energy supplied from the grid [kWh, calculated],
- *E*_tot batt ch_: total battery charge energy [kWh, from dataset],
- *E*_tot batt dch_: total battery discharge energy [kWh, from dataset],
- *E*_batt_: net battery energy [kWh, calculated],
- *E*_charger_: charger energy loss [kWh, calculated],
- *E*_aux_: energy attributed to auxiliary devices and other minor losses [kWh].

**Fig. 5:**
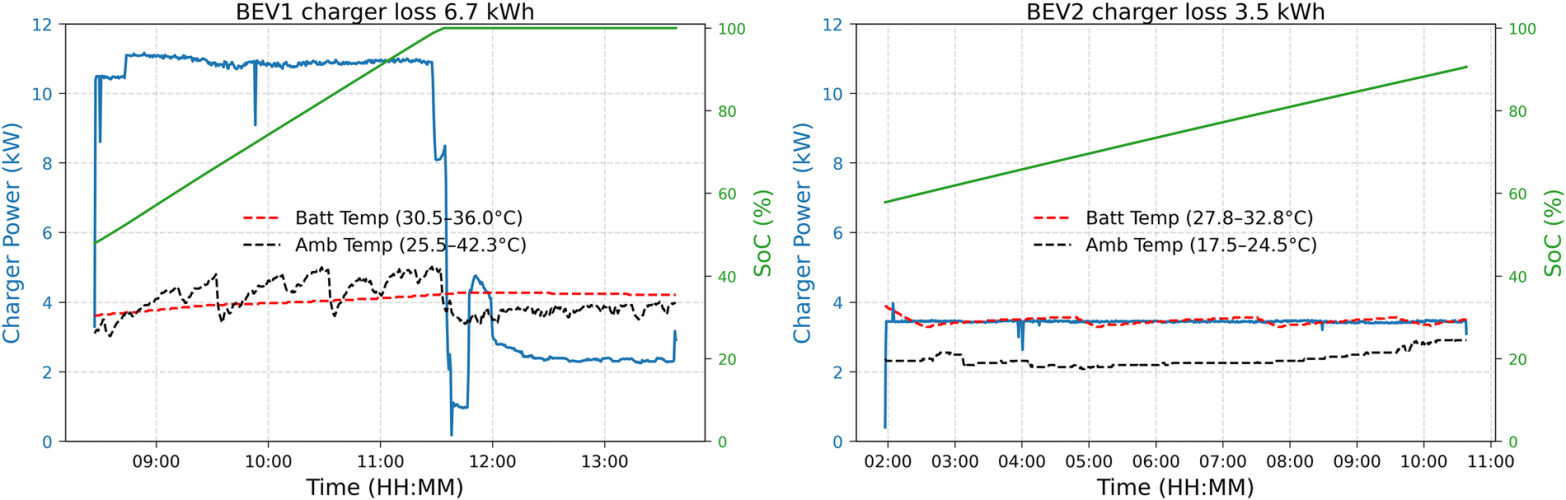
Two sessions with the highest charger energy losses.

Figure 5 illustrates charging profiles for the two sessions with peak charger loss. It can be seen that the grid continues supplying power at a lower level even though the SoC for BEV1 reached 100% (left subplot). This energy, which does not go to the battery, is most probably used to maintain the battery temperature, considering the higher environmental temperature (up to 42 °C). Combining this cooling energy requirement with a lower charging power level has increased the overall losses, resulting in 6.7 kWh out of 38.8 kWh supplied by the grid. This energy loss is around 17%. On the other hand, the losses can be mostly from the charger due to a lower charging power (3.4 kW) operation of BEV2, as shown in the same figure (right subplot). This lower power charging resulted in a 3.5 kWh loss out of 29.84 kWh supplied by the grid, which is around 12%. This energy dynamic clearly shows that vehicle thermal management settings and ambient temperature effects need to be considered for realistic grid impact modeling.

**Fig. 6:**
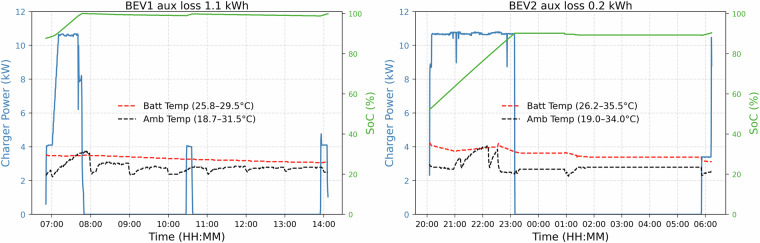
Two sessions with the highest auxilary energy losses.

Energy attributed to auxiliary devices is energy from the battery after the maximum SoC is reached. This energy is used by auxiliary devices. Charging sessions with this higher energy loss are depicted in Fig. 6. It can be seen that BEV1 reached 100% before 08:00. However, the SoC decreases slightly, which is why there are two short charging scenarios (around 10:30 and 14:00) to keep the SoC to 100%, as set by the user. A similar scenario can be seen for BEV2 users with 90% as the maximum SoC around 23:00.

**Fig. 7:**
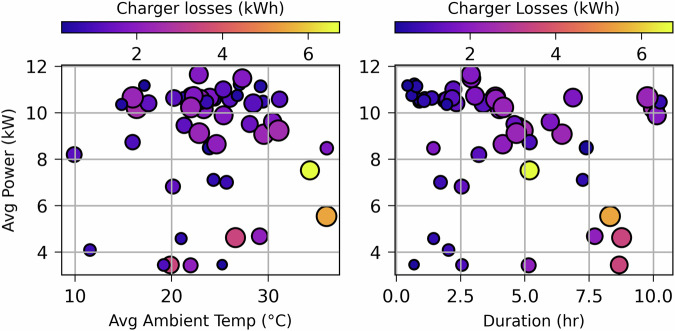
Charger losses in relation to ambient temperature, duration, and average power.

Figure 7 presents the charger losses observed in different charging sessions for both BEVs, highlighting their relationship with average charging power, ambient temperature, and session duration. The left subplot plots average ambient temperature against average charging power, while the right subplot correlates charging duration with average power. In both plots, the color scale represents charger energy losses and the size of each data point reflects the total battery energy charged during the session. It is evident that sessions characterized by lower average charging power (<8 kW), higher ambient temperatures (>30 °C), and extended durations (>5 hours) are associated with significantly higher charger losses.

### Fast Charging Data

**Fig. 8:**
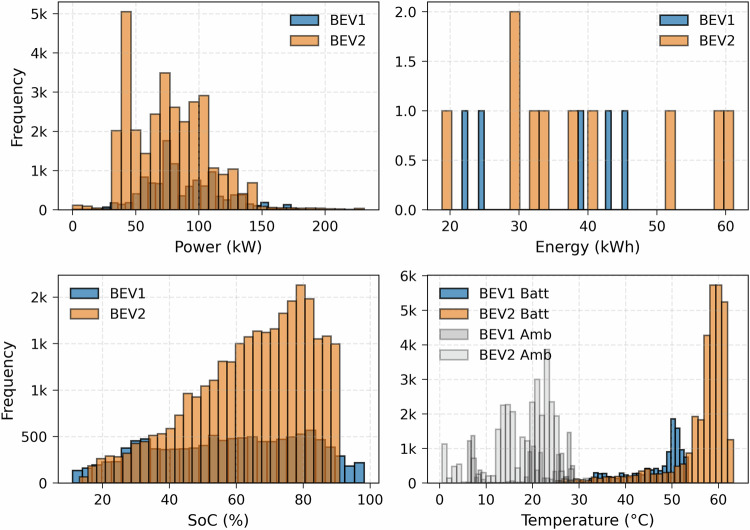
Fast charging dataset distribution.

The dataset collected from the two BEVs for fast charging sessions is illustrated in Fig. 8. This dataset includes public fast chargers using 150 kW and 250 kW systems. This histogram represents the distribution of charging data points in terms of charging power, session total energy, the SoC, and the battery maximum temperature. The BEV Amb temperature of charging stations is also shown. In fast charging, the charging power starts at its peak value and decreases slowly following the battery technology charging curve and operating conditions. The power distribution shows a higher frequency in the range of 40-150 kW, indicating the short time operation around the peak power values of chargers. The energy distribution shows the energy requirement for the two BEVs ranging from 20-60 kWh. This total energy requirement depends on the battery SoC at the beginning of the charging session. The SoC distribution is from 20–90% for BEV2 and 15–100% for BEV1. The BEV batt temperature distribution shows the temperature profiles during charging sessions, which go up 50 °C for BEV1 and 60 °C for BEV2, illustrating the heat generation during higher power operation.

**Fig. 9:**
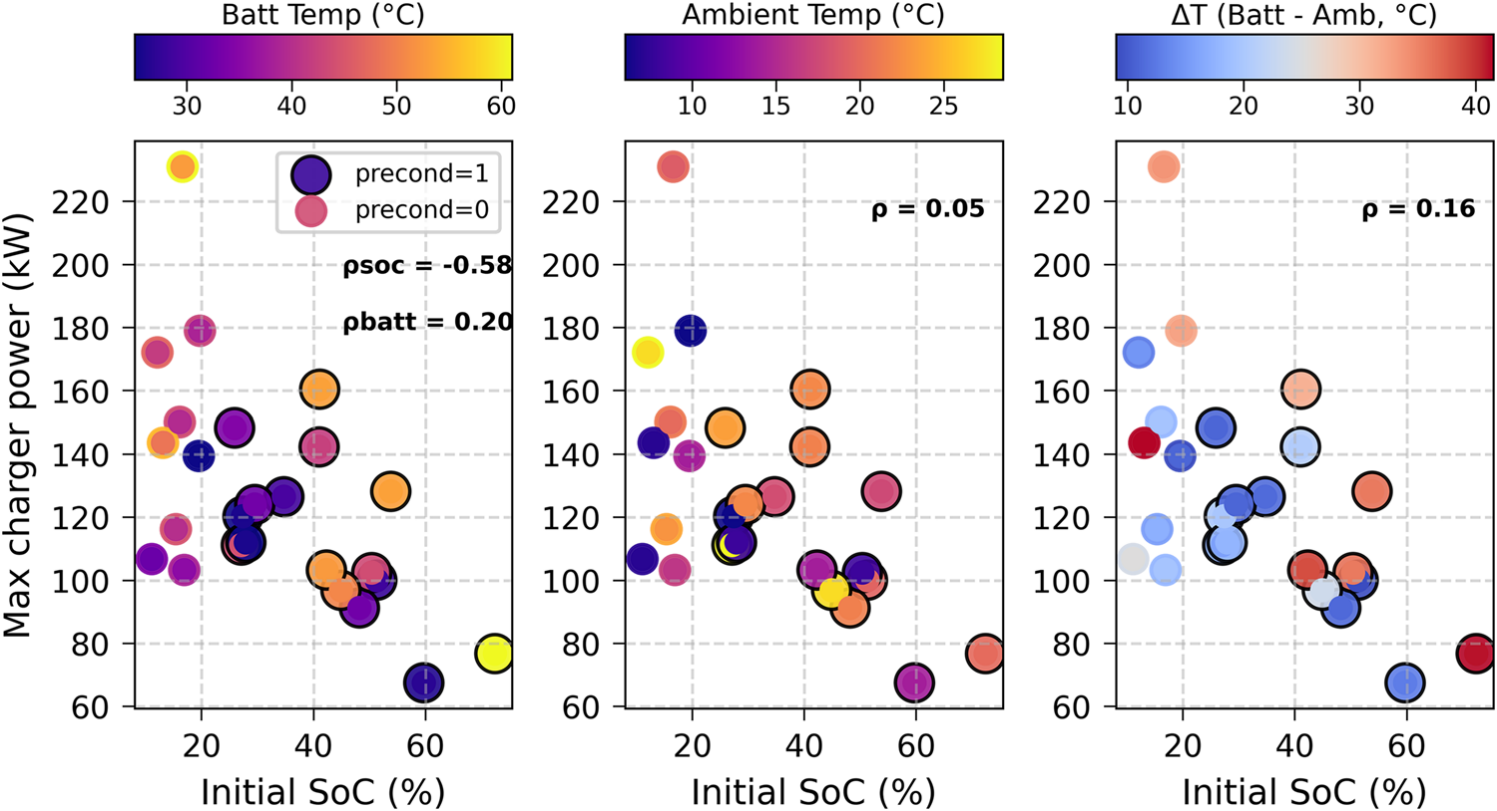
Maximum power in relation to the initial SoC, battery and ambient temperature.

The battery management system (BMS) and the charging station limit the charging power during charging sessions. The BMS is designed to limit the power based on the battery temperature and the arrival SoC, as illustrated in Fig. 9. This figure shows the charging sessions with battery preconditioning activated (precond=1) before the charging and those without preconditioning activated (precond=0). It can be seen that sessions starting at lower SoC have higher charging power, as indicated by the Pearson negative correlation (−0.58). At the same time, there is a weak correlation (0.2) between this power and the battery temperature. This correlation is much weaker with ambient temperature. The sessions with preconditioning were supposed to operate at higher power, but this was not always possible, which means that even if the fast charger and the BEV are capable of higher charging power, the operating conditions, such as the SoC and the battery temperature are limiting factors for this power.

**Fig. 10:**
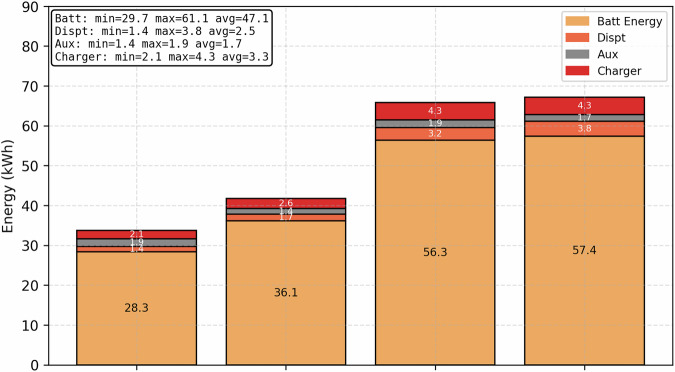
Fast charging energy breakdown.

The energy breakdown of some fast charging sessions with AC input energy data is illustrated in Fig. 10. The average energy loss collected from the 150 kW charger^15^ is around 3.3 kWh for these sessions, while the energy used by auxiliary devices and dissipation (Dispt) combined is in the range of 5 kWh. This means there are more losses from the vehicle than the charger in fast charging systems compared to slow charging. The overall energy losses range from 9%–12% for these charging sessions. It can be seen that charger loss and energy dissipation increase as the energy requirements increase while the auxiliary consumption stays in the range of 1.4-1.9 kWh. More fast charging datasets can provide better energy loss estimation.

### Driving Data

**Fig. 11:**
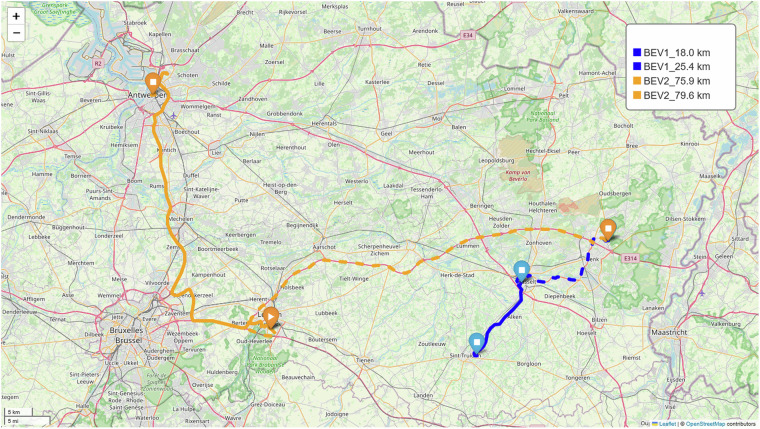
Main driving routes for BEV1 and BEV2 users.

The dataset was collected in Belgium from two BEV users with distinct usage profiles. Figure 11 illustrates the two most frequently traveled routes for each BEV, reconstructed from GPS trajectory data, including the trip distance for both users. As indicated in Table 3, BEV2 is equipped with a higher-capacity battery and is used for longer-distance mobility, frequently around 150 km/day. In contrast, BEV1 exhibits shorter daily driving distances, generally around 40 km/day, reflecting a more localized mobility pattern.

**Fig. 12:**
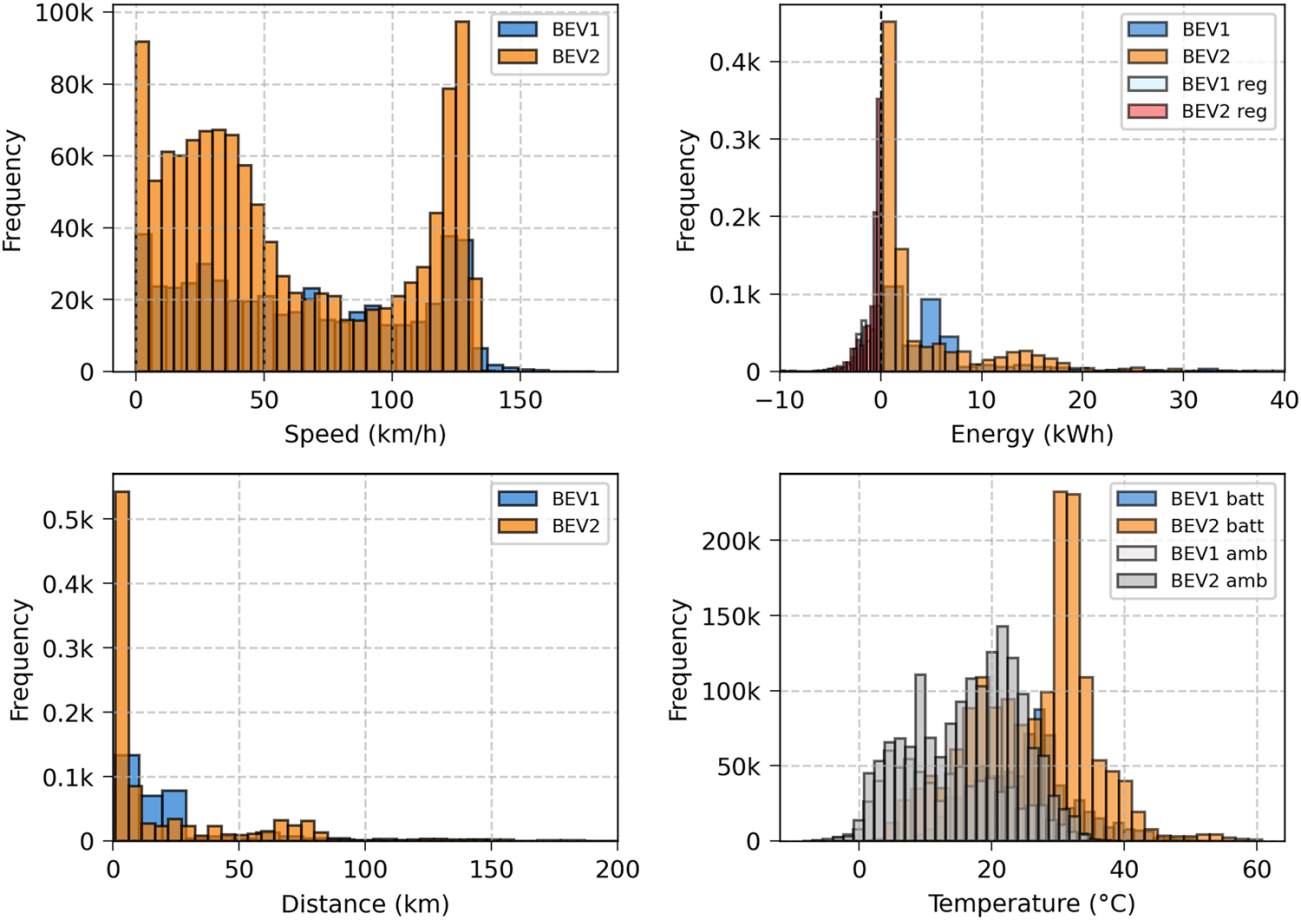
Driving dataset distribution.

Figure 12 illustrates the driving dataset distribution, including the vehicle speed, energy per trip, driving distance, and temperature. The top-left subplot shows the speed distribution, where both BEVs exhibit a bimodal pattern with peaks near urban (>50km/h) and highway speeds (125km/h). The top-right subplot illustrates energy used during driving and regenerative braking events (BEV reg). Most driving energy is below 10kWh, with BEV2 showing more high-energy discharges.

This regenerative braking in both BEVs is configured in Standard mode, enabling maximum possible energy recovery when the accelerator is released. However, the amount of this energy is limited under certain conditions such as higher SoC (95%), lower battery temperature, etc.

The bottom-left subplot illustrates the trip distance distribution, showing that most trips are short (<20km), with BEV2 performing more long-distance trips. The bottom-right subplot presents temperature distributions for the battery and ambient environment. BEV2 operates at higher average battery temperatures, with a peak frequency of around 32 °C, whereas BEV1 operates within a slightly lower thermal range.

**Fig. 13:**
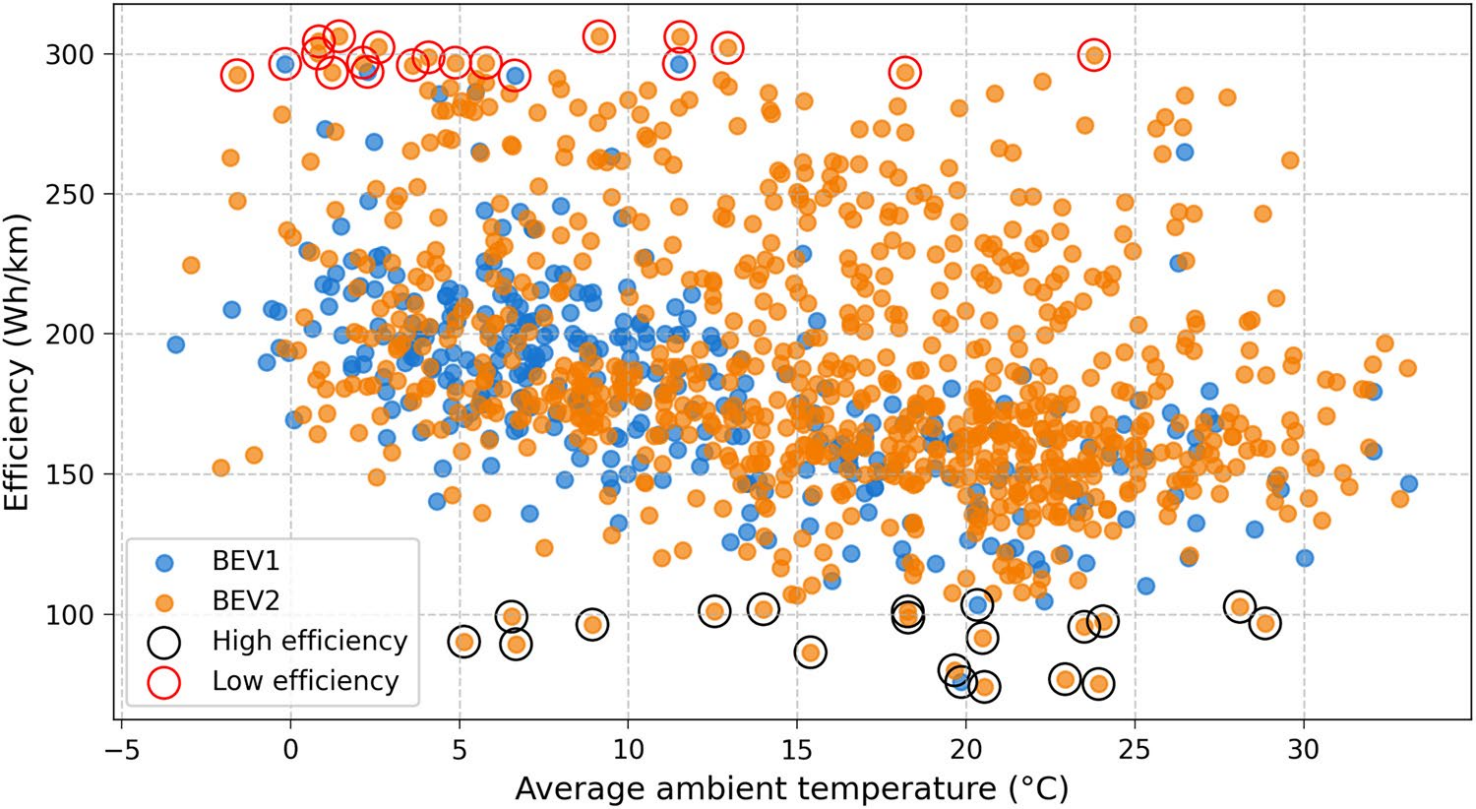
Relationship between average ambient temperature and driving efficiency.

Figure 13 illustrates the relationship between average ambient temperature and driving efficiency for the two BEVs. Each point represents a single driving session. The plot reveals a moderate trend where efficiency is generally higher at around 20 °C ambient temperature. Notably, driving sessions circled with a red edge indicate poor efficiency (around 300 Wh/km), often occurring at low ambient temperatures, possibly due to increased energy demands for battery heating, cabin conditioning, or driving behavior. In contrast, sessions circled in black edge demonstrate excellent efficiency (around 80-120 Wh/km), typically clustered at moderate temperatures between 15 °C and 25 °C. BEV2 exhibits greater variability and slightly higher consumption across the temperature range, while BEV1 appears more consistent. This illustration emphasizes the impact of ambient temperature on BEV efficiency and highlights possible best and worst-case performance conditions. This correlation with ambient temperature has direct implications for practical applications such as smart charging, where it can help improving demand forecasting; vehicle thermal management, by optimizing pre-conditioning and cooling strategies; and broader studies on range prediction and seasonal charging requirements.

Table 4 summarizes the driving efficiency statistics for the two BEVs. For each BEV, the number of valid driving sessions (N), the mean driving efficiency (Wh/km), the standard deviation (SD), and the 95% confidence interval (CI) are reported. BEV1 exhibited an average efficiency of 181.7 Wh/km across 333 sessions, with a SD of 35.2 Wh/km and a 95% CI ranging from 177.9 to 185.5 Wh/km. BEV2 showed a slightly higher average efficiency of 188.9 Wh/km across 854 sessions, with a SD of 46.4 Wh/km and a 95% CI of 185.8 to 192.0 Wh/km. The non-overlapping confidence intervals indicate that the difference in efficiency between BEV1 and BEV2 is statistically meaningful.

**Table 4:** Driving efficiency statistics for BEV1 and BEV2.

BEV	Number of sessions	Mean (Wh/km)	SD (Wh/km)	95% CI (Wh/km)
BEV1	333	181.7	35.2	177.9–185.5
BEV2	854	188.9	46.4	185.8–192.0

### Parking Data

**Fig. 14:**
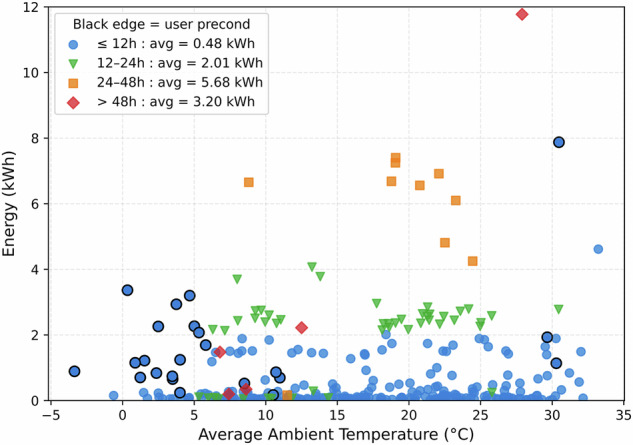
Energy consumption as a function of average ambient temperature during parking sessions.

Parking is defined as any time during which no charging or driving data was collected. During parking, energy is consumed for battery thermal management and auxillary devices used for communication, video recording, etc. Figure 14 illustrates the energy consumption as a function of average ambient temperature, clustered by parking duration intervals. Overall, the plot reveals that energy usage increases notably with parking duration. Four distinct marker styles represent these duration clusters.

Blue circles (12h) form a dense cluster at the bottom of the plot, showing consistently low energy usage with an average of 0.48 kWh. This group includes markers with black edges, indicating user-triggered preconditioning sessions, particularly during cold weather. The two blue circles with energy values higher than 4 kWh are from the summer hot season with a significant difference between with and without preconditioning. Green downward triangles (12-24h) exhibit moderate energy consumption scattered between 1-4 kWh, with an average of 2.01 kWh, appearing predominantly in the 5-25 °C range. Orange squares (24-48h) show a separated cluster with significantly higher energy, averaging 5.68 kWh, mostly concentrated between 17-25 °C. This group highlights the correlation between increased parking duration and higher energy usage. Red diamonds (>48h) present a small, more variable cluster with energy values spread across the full range, averaging 3.20 kWh. The outlier point, which reached up to 12 kWh, is long-time parking during summer break.

### Total Energy Data

**Fig. 15:**
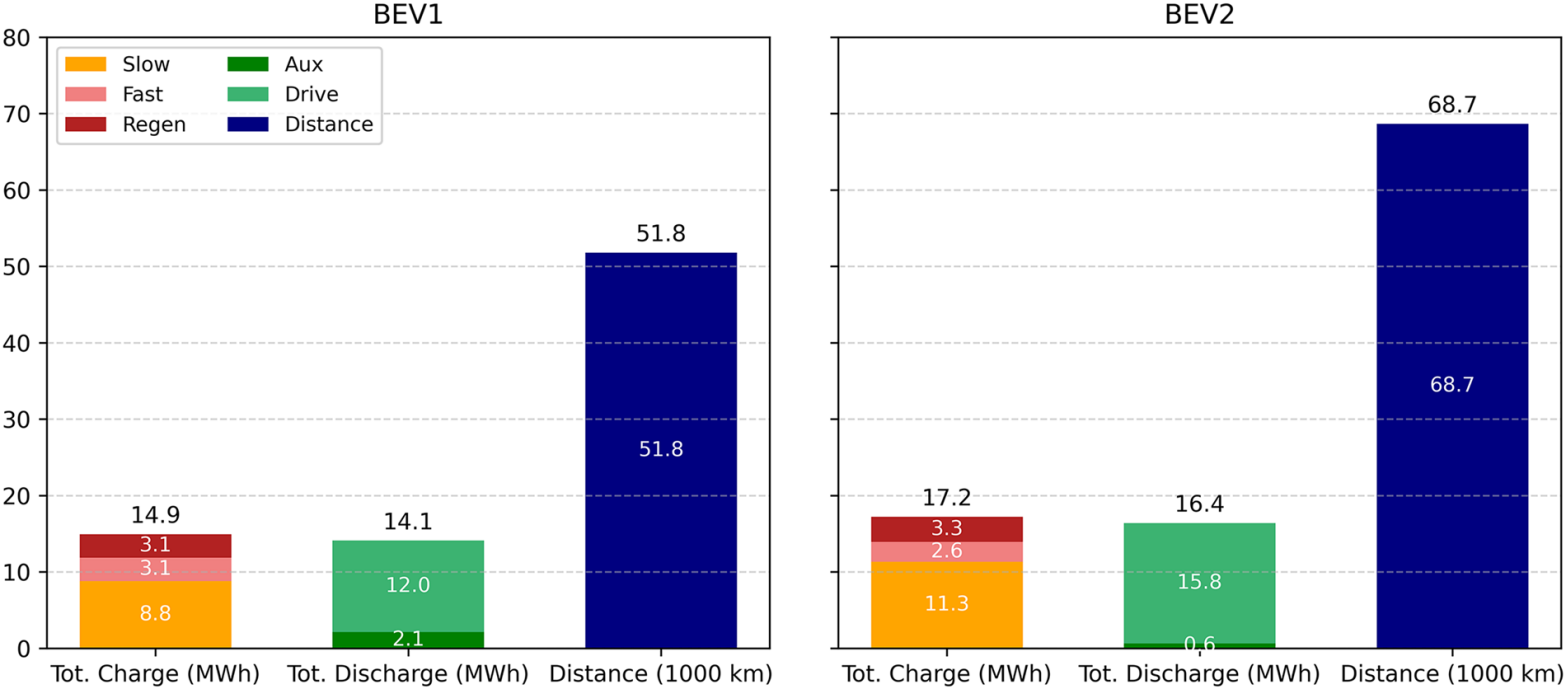
Total energy consumption for BEV1 and BEV2 during two years of operation.

Figure 15 compares the two BEVs’ total energy consumption and driving distance over a two-year operational period. Each bar group shows the total energy charged, total energy discharged, and total driving distance. For BEV1, the vehicle was charged 14.9 MWh, with 8.8 MWh from slow charging, 3.1 MWh from fast charging, and 3.1 MWh recovered through regenerative braking. The total discharge was 14.1 MWh, of which 12.0 MWh was used for driving and 2.1 MWh for auxiliary systems. The vehicle covered a total distance of 51.8 thousand kilometers.

In contrast, BEV2 recorded higher energy consumption and driving. It was charged with 17.2 MWh in total, comprised of 11.3 MWh from slow charging, 3.3 MWh from fast charging, and 2.6 MWh from regenerative energy. BEV2 discharged 16.4 MWh, with 15.8 MWh attributed to driving and 0.6 MWh to auxiliary loads. The total driving distance reached 68.7 thousand kilometers.

Overall, the figure illustrates that BEV2 (238 Wh/km) not only had greater energy throughput compared to BEV1 (272 Wh/km) but also higher efficiency in energy utilization for propulsion, as reflected in its greater distance traveled and higher drive energy relative to auxiliary use. It is worth noting that both BEVs are privately owned vehicles, primarily used for commuting by a single driver, and their tire pressures are typically around 3 bar, in line with the manufacturers’ recommendations.

This disparity in total energy consumption can be attributed to several factors related to both the BEV models and their operating conditions. BEV1 is LFP battery, while BEV2 employs a NCA battery. Although NCA battery provides higher energy density, it requires stricter thermal management, which increases auxiliary energy demand. BEV2 was also used more frequently for longer highway trips, resulting in higher average speeds and therefore greater aerodynamic losses. In addition, auxiliary usage, particularly HVAC, was more frequent and intensive for BEV2, further contributing to its higher energy consumption. Furthermore, the dataset for BEV2 is larger than that of BEV1, which makes a direct one-to-one comparison between the two BEVs more challenging.

## Data Records

The present dataset is publicly available in the KU Leuven research data repository under the CC BY 4.0 license (10.48804/8KPDTW). The dataset is organized in folder containing slow and fast charging, driving, and parking sessions. There are, in total, around 2310 files, of which 25 files are from fast charging, 109 files are from slow charging, 780 files are from parking, and 1396 files are from driving. Each CSV file from these folders is named as the specific BEV, the respective data collection date, and the operation mode, e.g. BEV1_ 2025-02-15_drive. Table 5 briefly describes each variable or column name in the CSV files. Additional files shared in the repository include:

- An example of a raw file
- An example of CSV file containing list of all the possible variables from the raw file
- An example of a preprocessed CSV file
- A DBC file
- Python code to process the data
- Python code to visualize the figures presented in this paper
- A README file
- A text file containing all the required Python libraries for processing and visualization
- An additional folder containing files for different total energy consumption analyses

**Table 5:** Short description of different data variables.

Column name	Description	Unit
BattVoltage132	Battery voltage values recorded during all the sessions	V
BMS_kwhDriveDischargeTotal	Battery drive total charge values recorded during driving sessions	kWh
BMS_kwhRegenChargeTotal	Battery regenerative total charge values recorded during driving sessions	kWh
BMS_maxDischargePower	Battery maximum discharge power values recorded during driving sessions	kW
BMS_maxRegenPower	Battery maximum regenarative power values recorded during driving sessions	kW
BMS_preconditionAllowed	Battery precondition status (0 or 1) recorded during all the sessions	bit
BMSdissipation312	Battery dissipation power values recorded during all the sessions and particularly higher and more useful for fast charging sessions	kW
BMSmaxPackTemperature	Battery maximum temperature values recorded during all the sessions	°C
BMSminPackTemperature	Battery minimum temperature values recorded during all the sessions	V
ChargeLineCurrent264	Grid AC current values recorded during slow charging sessions	A
ChargeLinePower264	Grid AC power values recorded during slow charging sessions	kW
ChargeLineVoltage264	Grid AC voltage values recorded during slow charging sessions	V
DI_uispeed	Speed measurement recorded during driving sessions	km/h
FC_dcCurrent	Fast charger DC current values recorded during fast charging sessions	A
FC_dcVoltage	Fast charger DC voltage values recorded during fast charging sessions	V
FCCurrentLimit244	Fast charger DC current limits values recorded during fast charging sessions	A
FCMaxCurrentLimit541	Fast charger maximum DC current limits values recorded during fast charging sessions	A
FCMaxPowerLimit541	Fast charger DC power limits values recorded during fast charging sessions	kW
FCMaxVlimit244	Fast charger maximum DC voltage values recorded during fast charging sessions	V
FCMinVlimit244	Fast charger minimum DC voltage values recorded during fast charging sessions	V
Odometer	Distance measurement recorded during driving sessions	km
RawBattCurrent132	Battery current values recorded during all the sessions	A
SOCave292	Battery average SoC values recorded during all the sessions	%
Timestamp	Timeseries data containing ISO 8601-formatted datetime values with UTC time zone	second
TotalChargeKWh3D2	Battery total charge values recorded during all the sessions	kWh
TotalDischargeKWh3D2	Battery total discharge values recorded during all the sessions	kWh
UI_Range	User interface range values recorded during all the sessions	km
UI_batteryPreconditioningRequest	User battery precondition request status (0 or 1) recorded during all the sessions	bit
VCFRONT_tempAmbient	Ambient temperature values recorded during all the sessions from the front vehicle controller	°C
VCRIGHT_tempAmbientRaw	Ambient temperature values recorded during all the sessions from the right vehicle controller	°C

## Technical Validation

In this section, the collected data is validated using data values collected by other off-vehicle systems, such as AC charging points (CP) and DC fast chargers (FC). Table 6 compares energy consumption between the CP^16^ and the values recorded from the BEV during slow AC charging sessions across five charging sessions selected from the two BEVs. The values indicate a close match between the measured energy from CP and the recorded BEV energy for each session, with small differences due to probably minor losses, data resolution difference, resampling, rounding, etc. This difference can be higher with higher energy values (50 kWh). Table 7 compares energy consumption between the FC^15^ and the values recorded from the BEV during fast DC charging sessions across seven charging sessions selected from BEV2. The values indicate a much closer match between the delivered FC and recorded BEV energy for each session. The difference is still closer, even with the sessions with high energy values.

**Table 6:** Energy comparison for the slow charging.

CP energy (kWh)	11.89	14.85	23.96	41.61	52.05
**BEV AC energy (kWh)**	11.75	14.69	23.86	40.95	48.41

**Table 7:** Energy comparison for the fast charging.

FC DC energy (kWh)	31.62	49.18	62.80	39.21	41.45	60.58	61.46
**BEV DC energy (kWh)**	31.66	49.20	62.78	39.16	41.43	59.97	61.50

It is worth noting that the final energy values depend on both the accuracy of the measurement devices for voltage and current, as well as potential errors in CAN bus frame recording. While BEV manufacturers do not disclose the specific sensing technologies integrated into their vehicles, the global accuracy of automotive-grade voltage and current sensors is typically within  ± 0.5% to  ± 1%^17^. The CAN data record is received around 10 or 20 ms intervals depending on the type of message^18^. Additionally, to minimize timestamp synchronization issue, the data points under steady-state conditions and at a sufficiently high resolution were considered in the final calculation.

Table 8 compares driving distances measured by onboard odometers versus distances estimated via Google Maps using the GPS data for two BEVs across two primary routes illustrated in Fig. 11. The data indicates good agreement between actual odometer recordings and map-based estimations, with minor deviations likely due to route variations, traffic conditions, or GPS resolution.

**Table 8:** Distance comparison for the main driving routes.

BEV	Route 1		Route 2	
	**Odometer (km)**	**Google Map (km)**	**Odometer (km)**	**Google Map (km)**
BEV1	18.03	17.9–18.3	23.11	24.6–25.5
BEV2	63.89	64.1–69.3	79.71	77.5–79.6

**Fig. 16:**
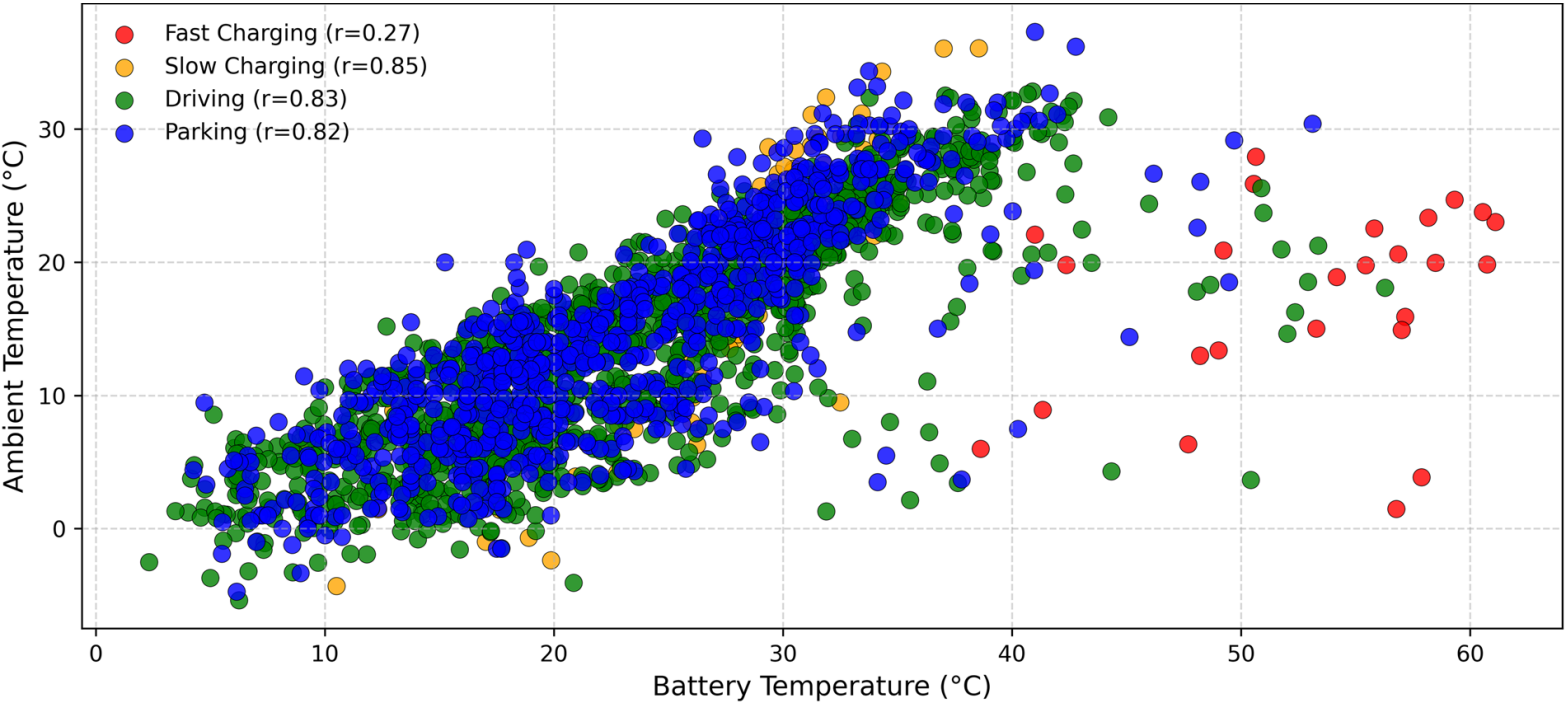
Correlation between the battery and ambient temperature over all the sessions.

Data are collected from two temperature sensors (vehicle controller right and front sensors), indicating the average values for ambient temperature during all the sessions. However, to illustrate the correlation between battery temperature and ambient temperature across all the sessions, averaged temperature values for each session are plotted in Fig. 16. It can be seen that a positive correlation (r=0.8) exists between battery temperature and ambient temperature in slow charging, driving, and parking sessions. This data indicates that battery temperature remains closely aligned with external conditions during these sessions, likely influenced by passive thermal behavior or limited heat generation at light and moderate power levels of operation during driving and slow sessions.

On the other hand, this correlation (r = 0.27) is weak during fast charging because the battery temperature becomes less dependent on ambient conditions at higher power charging operation. In this operation, the vehicle thermal management system actively regulates the battery temperature within a defined range as a function of power and SoC dynamics, as illustrated in Fig. 9. Both thermal inertia and hysteresis of lithium-ion batteries introduce a delayed response of temperature to power fluctuations, further reducing the zero-lag correlation. The dataset for fast charging sessions are relatively short in duration and fewer in number, as such events typically occur during long-distance or weekend trips.

## Usage Notes

All CSV files in the present dataset are provided in their final, processed form with a time resolution of one second, requiring no additional tools for further preprocessing. An example of a raw data file is also included to illustrate the extraction and preprocessing workflow. This workflow can be applied to similar datasets recorded from BEVs. Additionally, Python scripts are provided to reproduce various visualizations and analyses discussed in this paper.

## Data Availability

The dataset is openly available in the KU Leuven Research Data Repository under the Creative Commons Attribution 4.0 (CC BY 4.0) license at 10.48804/8KPDTW.

## Code availability

Python scripts for data processing and illustration are available in the dataset repository and on Github^19^. The required Python libraries are found in the additional files as pylibs.txt.
